# Application of targeted enrichment to next-generation sequencing of retroviruses integrated into the host human genome

**DOI:** 10.1038/srep28324

**Published:** 2016-06-20

**Authors:** Paola Miyazato, Hiroo Katsuya, Asami Fukuda, Yoshikazu Uchiyama, Misaki Matsuo, Michiyo Tokunaga, Shinjiro Hino, Mitsuyoshi Nakao, Yorifumi Satou

**Affiliations:** 1 Center for AIDS Research, Kumamoto University, Japan; 2 International Research Center for Medical Sciences, Kumamoto University, Japan; 3 Priority Organization for Innovation and Excellence, Kumamoto University, Japan; 4 Department of Medical Physics, Faculty of Life Sciences, Kumamoto University, Japan; 5 Department of Medical Cell Biology, Institute for Molecular Biology and Embryology, Kumamoto University, Japan; 6 Core Research for Evolutionary Science and Technology (CREST), Japan Science of Technology Agency, Tokyo, Japan

## Abstract

The recent development and advancement of next-generation sequencing (NGS) technologies have enabled the characterization of the human genome at extremely high resolution. In the retrovirology field, NGS technologies have been applied to integration-site analysis and deep sequencing of viral genomes in combination with PCR amplification using virus-specific primers. However, virus-specific primers are not available for some epigenetic analyses, like chromatin immunoprecipitation sequencing (ChIP-seq) assays. Viral sequences are poorly detected without specific PCR amplification because proviral DNA is very scarce compared to human genomic DNA. Here, we have developed and evaluated the use of biotinylated DNA probes for the capture of viral genetic fragments from a library prepared for NGS. Our results demonstrated that viral sequence detection was hundreds or thousands of times more sensitive after enrichment, enabling us to reduce the economic burden that arises when attempting to analyze the epigenetic landscape of proviruses by NGS. In addition, the method is versatile enough to analyze proviruses that have mismatches compared to the DNA probes. Taken together, we propose that this approach is a powerful tool to clarify the mechanisms of transcriptional and epigenetic regulation of retroviral proviruses that have, until now, remained elusive.

Exogenous retroviruses, such as the human immunodeficiency virus type-1 (HIV-1) and the human T-cell leukemia virus type-1 (HTLV-1), integrate into host genomic DNA in the form of a provirus. The provirus works as a DNA template for viral mRNA transcription to produce viral proteins, later assembled to form viral particles for *de novo* infection. In addition, the provirus is thought to play an important role in persistent retroviral infections, because it is neither targeted by the host immune system, which is composed of virus-specific cytotoxic T-lymphocytes and antibodies, nor removed by the currently available anti-retroviral drugs^1^.

HTLV-1 is the causative agent of adult T-cell leukemia (ATL), a leukemia of infected CD4^ + ^ T cells, and is also associated with several inflammatory diseases^2^,^3^,^4^. A unique characteristic of HTLV-1 is that the virus increases its copy number not via viral particle production or *de novo* infection, but via the proliferation of infected cells in which it remains as a provirus integrated into host genomic DNA^5^. Therefore, understanding the regulation of proviral transcription is key to understanding the pathogenesis of HTLV-1 infection, including the mechanisms leading to transformation of infected cells or the establishment of chronic inflammatory diseases. In contrast, HIV-1 replicates via vigorous production of viral particles. However, once HIV-1-infected individuals are treated with combination anti-retroviral therapy (cART), the plasma viral RNA load is reduced to undetectable levels in most infected individuals^6^. Thanks to recent advances in the development of anti-retroviral drugs, reducing the onset of AIDS in patients infected with HIV-1 is currently possible^7^. However, a problem that remains is that HIV-1 cannot be completely eradicated from infected individuals by existing cART. Efficient and highly sensitive experimental methods, applicable to the analysis of proviruses, are required to elucidate the mechanism of persistent HIV-1 infection under cART^6^.

Proviruses are integrated in the human genome, and are therefore chromatinized and regulated by the same epigenetic mechanisms acting on the human genome. To elucidate these regulatory mechanisms acting on the transcription of integrated proviruses, ChIP analysis is a useful experimental approach. In particular, ChIP-seq enables analysis of the epigenetic pattern at a very high resolution. However, a technical issue during ChIP-seq analysis of integrated proviruses is the low efficiency of proviral sequencing. This is because the size of a retroviral genome is very small: approximately 9,000 base pairs (bp) compared to the 3 billion bp of the human genome. In particular, when only analysis of the provirus is required, less than 0.01% of sequencing data is comprised of sequences from the proviral genome. In fact, we previously analyzed several epigenetic modifications of the HTLV-1 provirus using a ChIP-seq approach^8^. About 80 million reads were sequenced in the CCCTC-binding factor (CTCF)-ChIP-seq analysis, which detected in the order of 180 reads from the HTLV-1 genome. This was the motivation for us to develop a more sensitive and cost-effective sequencing method to analyze the retroviral genome integrated within host human genomic DNA.

In this study, we have established an efficient sequencing method that uses biotinylated DNA probes. It is a potentially powerful tool for retroviral latency research, such as studies of HTLV-1 and HIV-1 infection, by facilitating the analysis of epigenetic features, DNA-binding proteins, and deep sequencing of proviral genomes.

## Results

### Design of DNA probes and experimental flow for the enrichment of HTLV-1 proviral DNA

**Figure 1:**
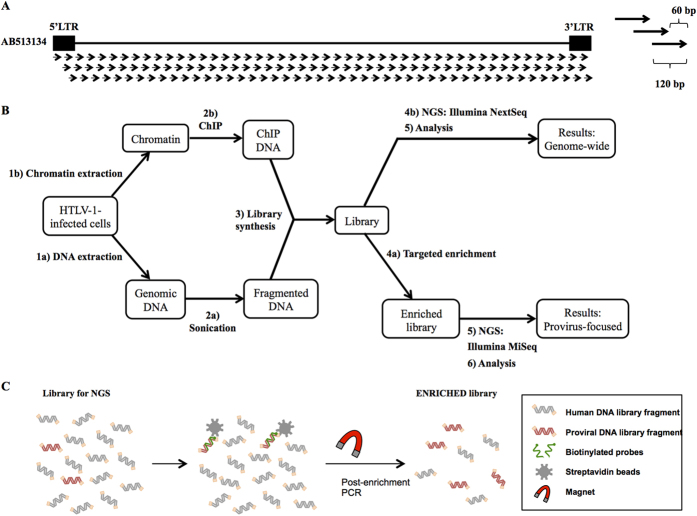
Design of DNA probes and experimental flow for the enrichment of HTLV-1 proviral DNA. (**A**) Design of the probes. One hundred and forty eight probes, 120 bp in length and with a 60 bp tiling, were constructed. (**B**) Experimental flow for the applications of the targeted enrichment. Genomic DNA or chromatin samples extracted from infected cells can be analyzed to render genome-wide or provirus-focused information. The enrichment of genomic DNA samples containing the viral genome may help discover new proviral sequences with more accuracy due to the higher number of reads that are obtained during the process. The enrichment of ChIP DNA could provide information on the epigenetic profile of the provirus. In addition, not enriching the samples could give the genome-wide context of the proviral profile. (**C**) Experimental flow for the enrichment protocol. DNA libraries prepared for NGS are mixed with the virus-specific biotinylated probes to allow the hybridization. Subsequently, streptavidin-coated magnetic beads are added to allow the isolation of the proviral DNA fragments.

We designed a set of DNA probes for the targeted enrichment of the proviral genetic material by hybrid capture. One hundred and forty-eight probes covering the entire provirus, 120 bp in length with a tiling of 60 bp, were designed based on the HTLV-1 reference sequence listed under accession number AB513134^9^ (Integrated DNA Technologies) (Fig. 1A). The probe-based capture method includes steps such as the preparation of fragmented DNA, library synthesis, an enrichment process, and next-generation sequencing (NGS) (Fig. 1B). The method may be applied to the analysis of integrated proviruses not only to determine their genomic sequence, but also to characterize their epigenetic profile. The enrichment step itself starts with a library prepared for NGS; i.e. adaptor sequences are linked to the DNA fragments (Fig. 1C). The library is mixed with human Cot-1 DNA and blocking oligos, before the addition of the virus-specific biotinylated probes, which are allowed to hybridize and capture their target sequences at 65 °C for four hours. Magnetic beads are added in order to easily separate the target DNA fragments, which have hybridized with the probes. After several washing steps, the DNA fragments of interest are eluted and PCR-amplified for NGS analysis. To test the targeted enrichment method, we analyzed the DNA of an HTLV-1 molecular clone, pACH^10^, mixed with human genomic DNA extracted from Jurkat cells (~4.5 ng of pACH DNA/1μg of Jurkat DNA). We analyzed the prepared library without enrichment in an Illumina NextSeq sequencing system, which rendered an average of around 120 × 10^6^ reads. Of these reads, 2 × 10^6^ mapped to the provirus, giving 1.9% provirus-specific reads per sample (Table 1). Next, we enriched the same libraries (250 ng of libraries prepared in duplicate, totaling 500 ng of starting input DNA for the enrichment step) and sequenced them in an Illumina MiSeq device. The proportion of viral sequences within the total number of reads was significantly increased from 1.9 to 99% (Table 1).

### Sequencing coverage of each proviral region in the enrichment protocol

**Figure 2:**
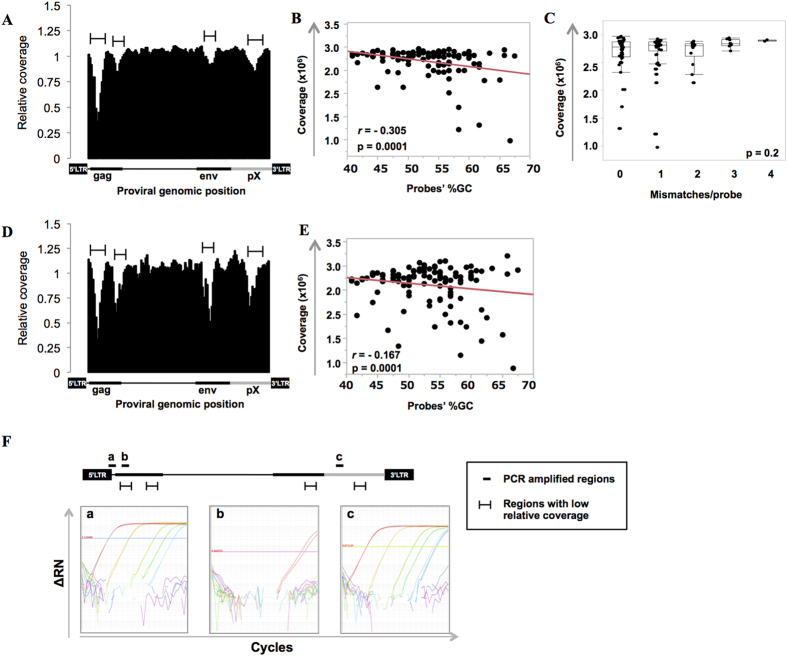
Evaluation of the library preparation and targeted-enrichment processes. The HTLV-1 molecular clone pACH was mixed with Jurkat cell line DNA and processed for high-throughput sequencing, with or without a targeted enrichment step. (**A**) Relative coverage values are shown that indicate how efficiently the target sequences were captured in samples undergoing enrichment. These were calculated as detailed in the Methods section. Bars indicate regions with low relative coverage values. (**B**) The influence of the probes’ GC content on the coverage and (**C**) the influence of mismatch number per probe on the coverage are shown. (**D**) For comparison, relative coverage values for the non-enriched sample were calculated. Bars indicate regions with low relative coverage values, and are the same regions shown in (**A**). **(E**) The influence of the probes’ GC content on the relative coverage is shown. (**F**) Quantitative PCR amplification plots of the indicated regions (a,b and c) within the proviral pACH sequence are shown. ΔRn is the magnitude of the signal generated (calculated as described in the Methods section) and plotted against PCR cycle number. Samples ranging from 10^8 ^copies/μl (red line) to 100 copies/μl (light blue line) were used. Regions that showed low relative coverage values in (**A**) and (**D**) are also indicated by black bars.

In order to thoroughly evaluate the efficiency of the targeted enrichment for each proviral region, we analyzed data from the mixed Jurkat cell line and pACH clone DNA (Table 1). The sequencing coverage is a measure of the number of reads that align to known reference bases. The cumulative coverage for each probe was calculated as the sum of the coverage for each base annealing to the probe. After calculating the mean of all probes, we compared each individual probe’s coverage to this mean value, to obtain the relative coverage value (Fig. 2A). This value for each probe was similar along the provirus, although it presented regions with low coverage (indicated by bars in Fig. 2A). Because targeted enrichment is based on the probe annealing to complementary sequences in the sample, factors such as the probes’ GC content or the presence of mismatches relative to the target sequence, might affect capture efficiency. Although a high degree of GC content showed a tendency to negatively influence the capture efficiency, a significant correlation was not observed (*r* = −0.305) (Fig. 2B). Furthermore, up to 4 mismatches per probe did not constitute a significant interference in the enrichment step (Fig. 2C). To distinguish whether or not the low coverage rate was caused by the enrichment step, we also analyzed the same library before enrichment. In order to facilitate the comparison, we analyzed both enriched and non-enriched samples by the same method and showed the data as “Relative coverage” per probe, although we didn’t use probes for the non-enriched samples. As shown in Fig. 2D, the general pattern in the coverage was similar to that of the enriched sample, including regions with a low coverage (indicated by bars in Fig. 2D), suggesting that the enrichment process was not the cause of low sequencing coverage in Fig. 2A. The library synthesis step included an amplification of the DNA fragments by PCR. It is known that factors, such as the GC content of a sequence, influence PCR amplification efficiencies^11^. In order to determine whether the low sequencing coverage was the result of poor PCR amplification, we performed a quantitative PCR (qPCR) analysis targeting the region that showed the lowest coverage, together with other proviral regions for comparison (Fig. 2F). We found that the PCR efficiency was remarkably lower in the region of low coverage compared to other proviral regions (5-LTR and pX region)^8^, indicating that differences in PCR efficiency could be a cause of variable sequence coverage.

### Capture of proviral sequences in HTLV-1-infected cells

**Figure 3:**
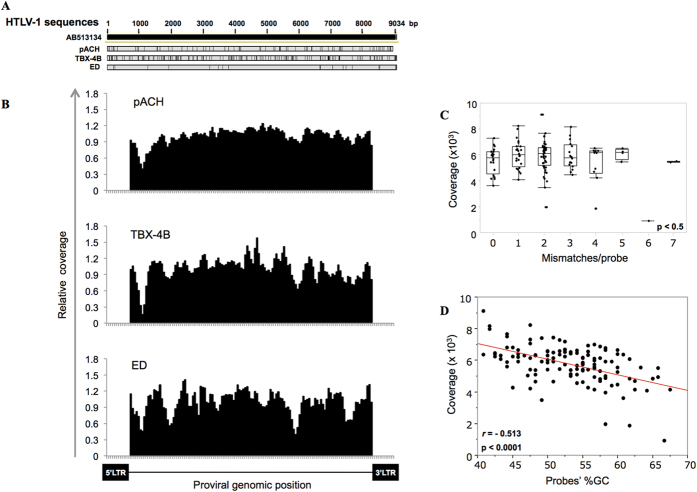
Capture of proviral fragments of HTLV-1-infected cells. (**A**) The effect of sequence variation on probe-enrichment efficiency was evaluated. A simplified comparative scheme of the analyzed HTLV-1 proviral sequences is shown. Vertical black lines indicate mismatches compared to the reference sequence (AB513134). ED is an ATL-derived T cell line, and TBX-4B is a non-malignant clone isolated from an HTLV-1-infected patient. (**B**) The relative coverage in the three analyzed proviral sequences is shown. (**C**) The analyses of the coverage in relation to the number of mismatches per probe or the GC content of the probes (**D**) are shown for the TBX-4B cell line, which presents a higher number of mismatches compared to the reference sequence.

In order to investigate the practical application of the enrichment method, we enriched DNA samples from two different HTLV-1-infected cell lines (ED and TBX-4B), in addition to the pACH clone and Jurkat cell DNA mixture prepared at the same molar ratio (5.4 pg of pACH DNA/1 μg of Jurkat DNA). The proviral sequences had a varying extent of mismatch to the reference sequence (GenBank, AB513134) (Fig. 3A). Despite the presence of mismatches, the relative coverage pattern of each capture was similar for the 3 proviral sequences, confirming that mismatches are of limited importance to capture efficiency (Fig. 3B). Plotting the number of mismatches in relation to the coverage for the TBX-4B cell line indicated that, at least in the range observed here (0 ~ 7), the number of mismatches per probe did not significantly affect the enrichment step (Fig. 3C). Consistent with the results in Fig. 2, the GC content did indeed show a tendency to negatively affect the capture efficiency, although still, the correlation between coverage and GC content was not significant (*r* = −0.513) (Fig. 3D).

### Enrichment of ChIP-seq libraries increases the sensitivity of proviral sequence detection

**Figure 4:**
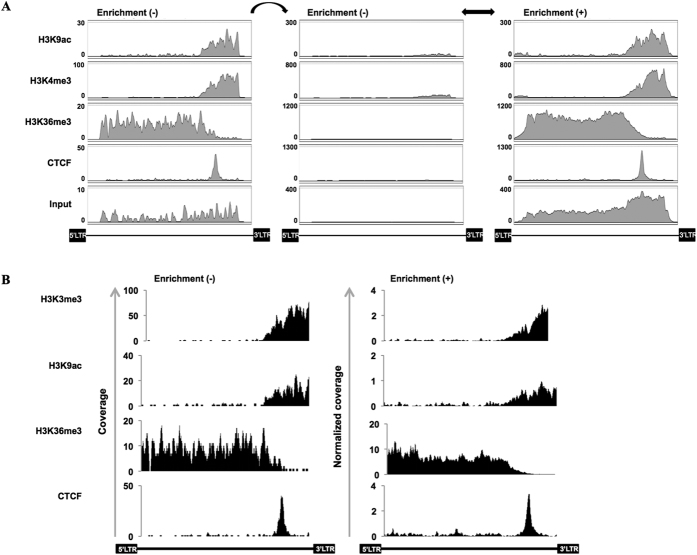
Application of targeted enrichment to ChIP-seq analysis. (**A**) ChIP-seq analyses for the indicated histone modifications were performed for the ED cell line. Libraries, before (left set of graphs) or after enrichment (right set), were subjected to high-throughput sequencing, and their profiles compared. Data for the non-enriched sample, obtained from the DNA Data Bank of Japan (accession no. DRA004162)^8^, are shown with two different scales to highlight the increase in reads after enrichment (left and middle sets). (**B**) The coverage of the enriched samples were normalized against the input DNA (right set of graphs) as described in the Methods section. These show a pattern similar to the non-enriched samples (left set).

To apply the enrichment method to ChIP-seq analysis of HTLV-1 provirus, we performed probe-based enrichment on previously generated ChIP-seq libraries. We recently reported that CTCF bound to HTLV-1 provirus and defined a border in the epigenetic modifications profile^8^. In this previous study, we sequenced ChIP-seq libraries without enrichment. Here we sequenced the same libraries after enrichment. Profiles of each mark for both types of samples, enriched and non-enriched, are shown in Fig. 4A. Although their epigenetic profiles retained a correlation, the number of reads mapping to the proviral sequence in the non-enriched sample was very low, compared to the total amount of reads obtained during the run (Table 2). This confirmed the scarcity of proviral fragments within the sample. In sequencing the enriched samples, the number of reads mapping to the provirus increased significantly, even though the total number of reads was much smaller compared to the non-enriched samples (Table 2). An increased number of hits to the proviral sequence provided higher resolution and solidity to the epigenetic pattern analyzed (Fig. 4A). Considering that performing a ChIP-seq analysis includes additional steps that might bias the overall procedure, we normalized the results of the ChIP samples to those of the Input sample; i.e., we divided the coverage of each base read in an individual ChIP sample, by the coverage value of the same base read in the Input sample. We observed the same epigenetic pattern as the non-enriched samples (Fig. 4B). Overall, these results suggest that enriching ChIP-seq libraries for proviral sequence analyses constitutes a reliable approach to analyze the epigenetic pattern of the integrated provirus, with high-throughput depth.

### Sequencing error rate of the NGS data with capture probes

**Figure 5:**
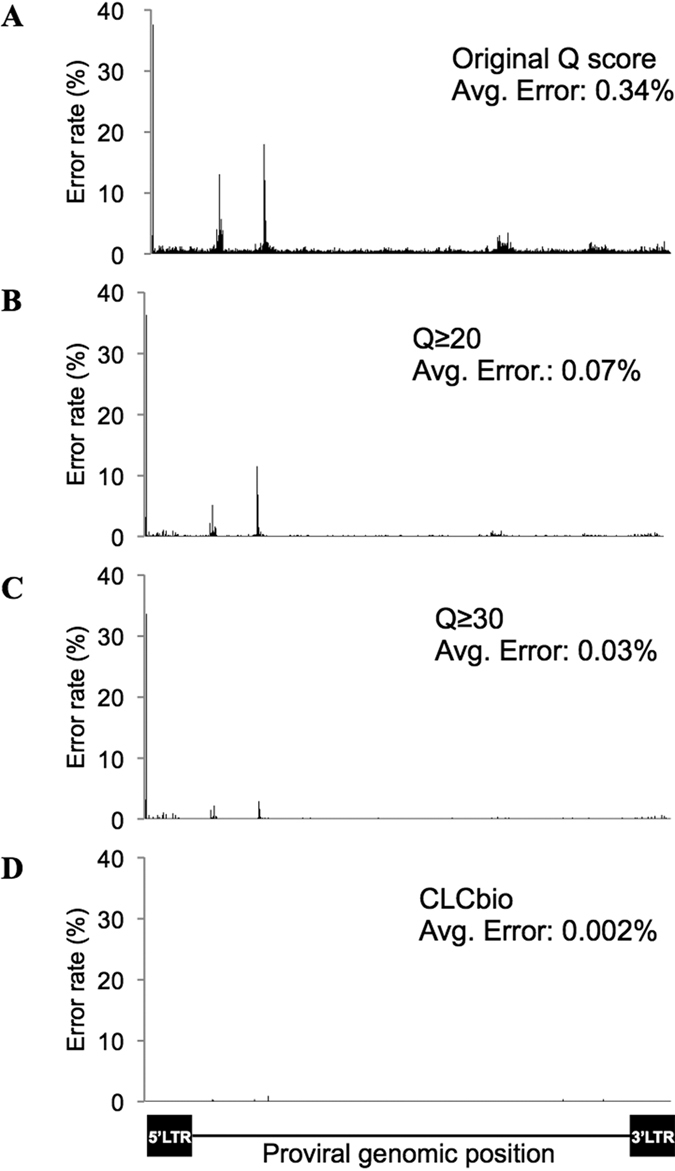
Sequencing error rate of the NGS data with capture probes. Error rates were calculated with different sequencing Q score settings. (**A**) Error rate for each base calculated with the default settings is shown. Increasing the cut-off values to 20 or 30 (**B** and **C**, respectively) led to a decreased error rate. **(D**) Error rate calculated with the software CLCbio is shown for the same sample.

In order to apply the enrichment method to the deep sequencing of viral genomes, we calculated the error rate of the protocol by again using the HTLV-1 molecular clone, pACH. There should be no variation or mutations in the viral sequence of this molecular clone. Thus, mismatches observed between sequencing data and the reference sequence should arise as a result of either the PCR amplification or sequencing steps. We performed only 6 cycles of PCR for library synthesis and an additional 10 cycles for amplification after the probe-enrichment step. The error rate for the PCR amplification should be very low with such a small number of PCR cycles. The high error rates we observed, as shown in Fig. 5A, are therefore likely to be generated by the NGS step.

Illumina sequencers are characterized by (i) solid–phase amplification, (ii) a cycle reversible termination process, and (iii) sequencing-by synthesis (SBS) technology^12^. It has been previously reported that certain DNA sequences trigger errors during the sequencing step^13^. We found that positions with high error rates were located at the end of a continuous sequence of cytosines. Considering the mechanism of the SBS technology used by Illumina sequencers, regions with several contiguous cytosines would be difficult to accurately detect because of phasing issues. This might explain the high frequency of errors when sequencing HTLV-1 proviruses. Furthermore, in order to evaluate the fitness of our sequencing quality control settings, we determined the sequencing error rate after setting the sequencing quality score (Q score) cut-off value to 20 or 30 (Fig. 5B,C). Increasing the Q score cut-off values, or performing the analysis with software such as the CLC Genomics workbench 7.5 (Fig. 5D), led to a decrease in the error rate, suggesting that sequencing errors could also be minimized in the analysis step, after the acquisition of the NGS data.

### Application of targeted-enrichment to the study of HIV-1 provirus

HIV-1 is another retrovirus that has far more variability in its genetic sequence than HTLV-1^14^. Recent studies have highlighted the importance of reservoir cells in HIV-1 infections that persist despite an effective therapeutic strategy^6^. To gain more insight into the mechanisms regulating persistent HIV-1 infection, a thorough analysis of the provirus inserted into the host genome may provide important clues. Therefore, we tested our probe-based capture on HIV-1-infected samples, as was done for HTLV-1.

**Figure 6:**
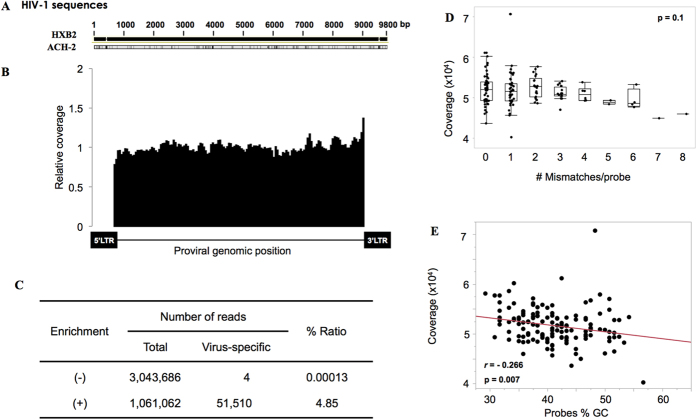
Application of targeted enrichment to the study of HIV-1 provirus. (**A**) Comparison of the HXB2 reference and ACH-2 cell line HIV-1 proviral sequences. Vertical black lines indicate mismatches compared to HXB2. (**B**) Relative coverage after enrichment of naked genomic DNA prepared for high-throughput sequencing. (**C**) Number of total and virus-specific reads obtained before and after enrichment of the libraries. Changes in the coverage, in relation to the number of mismatches per probe (**D**), or their GC content (**E**), are shown. The analysis was performed excluding the LTR portions of the sequence, as for the HTLV-1 provirus analysis.

Probes targeting the HIV-1 provirus were designed based on the HXB2 reference sequence (Fig. 6A), and then tested for capture using the genomic DNA of an HIV-1-infected cell line, ACH-2. Despite a great number of mismatches, the coverage analysis showed that the overall protocol was also effective for HIV-1 (Fig. 6B). The number of reads mapping to the provirus not only increased significantly after the enrichment step but, most importantly, the proportion within the total number of reads increased as well (Fig. 6C). Up to eight mismatches in a single probe did not significantly impair the capture (Fig. 6D), and a high percentage of GC content did not significantly correlate with a decrease in sequencing coverage, as observed for HTLV-1 (Fig. 6E). Thus, these results suggest that the probe-based capture could also be effective for the study of the HIV-1 provirus.

## Discussion

The advent of high-throughput sequencing technologies has had a significant impact on biological research. It has enabled the analyses of complex samples that were previously unsuitable for detailed study without significant investment of both money and time. They have been used to sequence the entire genome of several species, including the human genome within a few days, or to characterize variations between individuals of the same species^15^,^16^. Since the total length of the human genome is extremely long, over 3 billion DNA bp, the upfront cost of analyzing multiple samples by this approach is a prohibitive factor for many researchers. It has been possible though, to partially circumvent this problem by applying enrichment techniques, such as hybrid capture, selective circularization or PCR amplification^17^. Whole-exome sequencing is an application of NGS that can identify mutations or variations in the coding sequences of the human genome^18^. Approximately 30 million bp of the human genome (barely 1%) are coding sequences (the exome). It is possible to use a probe-based hybridization approach to enrich for exonic fragments, and this can increase the efficiency of exome-sequencing (exome-seq). There are currently several commercially available probe sets for exome-seq. Furthermore, probes have been previously used for the investigation of novel retroviruses associated with cancers^19^. However, there are no products for a probe-based enrichment of specific whole-retroviral genomes.

The genome size of a retrovirus is approximately 9 kb, making it suitable for this type of approach. In this study we therefore designed 148 and 161 probes in order to cover the entire HTLV-1 and HIV-1 genomes, respectively. We utilized xGen Lockdown Probes (IDT) that were individually synthesized probes for targeted enrichment by hybrid capture. In this study, we designed probes based on the sequence HTLV-1 subtype A or HIV-1 subtype B, because these strains are the most frequently found in Japan. Other sets of probes can be generated based on the sequence of other strains depending on the population to analyze^20^,^21^. We could generate, at one time, enough oligonucleotide probes to perform more than 100 targeted enrichment experiments, at a relatively low cost.

The enrichment of retroviral sequences allows for several possible applications. The first is ChIP-seq analysis to characterize molecules close to specific proviral sequences, as shown in this study. ChIP-seq analyses of cell lines containing one copy of the provirus, are a particularly suitable application, because there is no concern about sequence variation within the viral sequence. The probe-based enrichment method tested in the current study yielded a much stronger ChIP-seq signal than conventional ChIP assays performed without enrichment. In the latter, we needed to waste more than 99.99% of the sequencing power due to the overwhelming presence of sequences from the host human genome. We were able to increase the sensitivity of the ChIP-assay with a small number of sequencing reads by using the enrichment method. The second possible application is the deep sequencing of retroviruses. HTLV-1 is very stable in terms of viral sequence^14^, although there have been reports suggesting that some mutations play a role in the escape from host immune surveillance and contribute to leukemogenesis in ATL^9^,^22^. The conventional PCR-based protocol has several issues, such as the bias of PCR amplification, mutations in primer-binding sites, and the lack of information available on the initial sequences of the 5′LTR or the end of the 3′LTR. Thus, the probe-based enrichment method should be useful for deep sequencing because the mutation number would fall within the range tolerated for an efficient capture (up to 8 mismatches/probe). Although this method would show certain limitations for the analysis of samples containing a mixture of proviruses, decreasing the sequencing error rate, as we have shown in Fig. 5, would theoretically allow us to determine the sequences of several quasispecies with the obtained dataset. Based on an accurate base-calling step, the presence of discordant nucleotides in the same position within the provirus, combined with information on the number of proviruses present in the sample, could allow us to determine their sequence. The third application is integration-site analysis of retroviruses. A recent study reported the use of probes targeting the LTRs of subtype B HIV-1 strains^23^. This approach would be an alternative method for analyzing integration sites of HIV-1 or HTLV-1 in infected cells, in addition to a linker-mediated PCR protocol^24^,^25^,^26^,^27^. Randomly fragmented pieces of DNA containing the junction provirus-host DNA can be obtained and will be later captured by the probes targeting the LTRs. So, during the mapping step, one can determine the integration site within the human genome.

One of the major topics in HIV-1 research is latency under cART. In contrast to HTLV-1, the HIV-1 sequence is highly variable, making this approach unsuitable for the deep sequencing of its provirus. However, we have designed overlapping probes for the viral sequence, which reduces concerns about mismatches. In addition, this method could be applied to the analysis of HIV-1 RNAs present in plasma, because these are generally much less variable than the proviral sequence. For example, analyzing the changes in the viral sequence of the founder virus in an animal model infected with HIV-1 would be another suitable application for this new method. In such an instance, we would be able to analyze how the viral sequence evolves in the model from the initial phase of infection to the persistent or latent one. HTLV-1 is also known as a retrovirus that spontaneously achieves latent infection without the administration of anti-retroviral drugs, and long-lived infected clones sometimes cause leukemia. However, the precise mechanism of HIV-1 and HTLV-1 retroviral latency is still not fully understood. The novel method described in this study would be a very powerful tool to elucidate the regulatory mechanisms of proviral transcription, sequence variation, and retroviral latency by providing more precise and accurate information about provirus DNA binding proteins, histone proteins, and histone modifying molecules. We propose that this new provirus enrichment and sequencing technique is an efficient and comprehensive approach able to provide higher resolution to the analysis of integrated retroviruses. Furthermore, multiplexing the samples and pooling them for sequencing offers a significant reduction in the cost of this experimental approach.

## Methods

### DNA probes

One hundred and forty eight xGen Lockdown Probes (Integrated DNA Technologies) were custom-designed based on the HTLV-1 proviral sequence, accession number AB513134 (the entire list of probes can be found as Supplementary Table S1). For the enrichment of libraries containing HIV-1 sequences, we designed 161 xGen Lockdown probes based on HXB2, a reference sequence for HIV-1 subtype B (GenBank, K03455) (the entire list of probes can be found as Supplementary Table S2). The biotinylated DNA probes targeting the entire proviruses are 120 bp in length with a 60 bp tiling, and were received dried in a single tube. Upon receipt, they were dissolved in a Tris-buffered solution (10 mM Tris/HCl pH 8.0, 0.1 mM EDTA) to a final concentration of 1.5 pmol/μl and stored at −20 °C until use, as recommended by the manufacturer.

### Cell lines and a plasmid

Two HTLV-1-infected cell lines were used. ED is an adult T-cell leukemia-derived cell line^28^, while TBX-4B is a non-malignant T-cell clone isolated from the peripheral blood of an HTLV-1-infected patient^29^. Both cell lines contain one copy of the integrated provirus. In addition, the cell line ACH-2, latently infected with HIV-1, was obtained through the AIDS Research and Reference Reagent Program, Division of AIDS, NIAID, NIH: ACH-2 from Dr. Thomas Folks^30^. To analyze the error rates of the sequencing step after probe-enrichment, we used an HTLV-1 molecular clone pACH^10^. ED and ACH-2 cell lines were cultured in RPMI supplemented with 10% FBS, 100 U/ml penicillin and 100 μg/ml streptomycin. The TBX-4B cell line was cultured in RPMI supplemented with 20% FBS, penicillin, streptomycin, and 200 U/ml of recombinant IL-2 (Wako).

### Sample preparation

DNA extraction was performed using the DNeasy Blood and Tissue kit (Qiagen). DNAs were fragmented to an average length of approximately 300bp using Covaris S220 (Covaris) or Bioruptor USD-300 (Diagenode) sonication devices. Chromatin was extracted by cross-linking cells in a 1% formaldehyde solution for 5 minutes at room temperature. After the addition of a glycine solution to a final concentration of 125 mM, cells were lysed in two consecutive steps to isolate nuclear extracts, which were then subjected to sonication to obtain DNA fragments of approximately 350 bp in length. Chromatin immunoprecipitation assays were performed as previously described^8^. The following antibodies were used: anti-human CTCF (07-729, Millipore), anti- H3K4me3 (07-473, Millipore); anti-H3K9Ac (17-658, Millipore); anti-H3K36me3 (ab9050, Abcam); and rabbit polyclonal control IgG (ab46540, Abcam).

### Library synthesis and high-throughput sequencing

DNA libraries required for high-throughput sequencing were prepared from fragmented DNA, or DNA obtained after ChIP, using the NEBNext Ultra DNA Library Prep Kit and NEBNext Multiplex Oligos for Illumina (New England BioLabs). Multiplexed libraries were subjected to cluster generation using a MiSeq Reagent Kit v3 (150 cycles) or NextSeq 500 Kit (75 cycles) in MiSeq or NextSeq desktop sequencing systems (Illumina), respectively.

### Targeted enrichment

After library synthesis, up to 6 samples, containing different Index sequences, were mixed together in order to reach the recommended amount of 500 ng, to perform the enrichment step involving hybridization with the virus-specific probes. This was done using the SeqCap EZ Hybridization and Wash Kit (Roche NimbleGen) following instructions of the rapid protocol for DNA probe hybridization and target capture from IDT. Briefly, library DNA was first mixed with 5 μg of human Cot-1 DNA (Invitrogen) and xGen Universal Blocking Oligos (IDT) and dried up using an evaporator (Eyela). The dried DNA was the dissolved in the hybridization buffer. After an incubation step of 10 minutes at 95 °C, the probes were added and hybridization was allowed to take place at 65 °C for 4h. Streptavidin-coated magnetic beads (Life Technologies) were added to the hybridization mixture, and the sample was additionally incubated for 45 minutes at 65 °C. After the recommended washing steps, the captured DNA was amplified by PCR and further purified using Agencourt AMPure XP beads (Beckman Coulter). DNA libraries enriched for proviral sequences were quantified by qPCR using Illumina’s P5 and P7 primers prior to the sequencing step.

### Data analysis

After obtaining Fastq files from the MiSeq or NextSeq systems, we performed an adaptor-trimming step and removed low-quality sequencing reads. The cleaned sequencing reads were mapped to the HTLV-1 (Genbank, AB513134.1) or HIV-1 genomes (Genbank, K03455) with or without the human reference genome (hg19) using the BWA-MEM algorithm^31^. We also analyzed ChIP-seq data of ED cells we previously deposited in the DNA Data Bank of Japan (accession no. DRA004162)^8^. PCR duplicates were removed with the Picard tool (http://broadinstitute.github.io/picard/). Mapped data were visualized by Strand NGS (Strand Life Science). The efficiency of the enrichment process was determined by comparing the number of reads mapping to the provirus relative to the total number of reads obtained after the analysis. The ratio (% ratio) was calculated as: number of virus-specific reads/total number of reads ×100. In order to determine the capture efficiency of each probe, the relative coverage values were calculated as the ratio between each probe’s cumulative coverage and the mean cumulative coverage of all the virus-specific probes. [Relative coverage for probe *X* = probe *X* cumulative coverage/mean cumulative coverage of all the probes].

To calculate the error rate of the sequencing step, we individually analyzed the sequencing quality score (Q score) of each base read, and identified bases with Q scores lower than 20 or 30. These bases were not removed. Instead, they were made to appear as “N”, leaving the coverage value intact, thus minimizing the loss of sequencing information. We also performed an error correction by average Q score values for each reference sequencing position by using the Low Frequency Variant Detection tool with the CLC Genomics workbench 7.5 (CLC Bio) following the manufacturer’s recommendations. It is worth noting that NGS data analysis was performed excluding the LTR portions of the sequence. Since both LTRs share the same nucleotide sequence, it is not possible to specifically determine which LTR a read mapping from this region corresponds to.

### Quantitative PCR (qPCR)

In order to test the bias that PCR amplification might exert on the preparation of the sample for targeted enrichment, a qPCR was performed using Thunderbird SYBR mix (TOYOBO) and the StepOnePlus Real Time PCR System (Applied Biosystems). The following primers were used: for region “a” in Fig. 2F: 5′-GACAGCCCATCCTATAGCACTC-3′ and 5′-CTAGCGCTACGGGAAAAGATT-3′; for region “b”: 5′-CATACTCATCCAAACCCAAGC-3′ and 5′-GGTGCATGACTGGAAGGACT-3′ or 5′-ATCCAAACCCAAGCCCAGAT-3′ and 5′-GGGCCGTAGGCTCAACATAG-3′; for region “c”: 5′-CTGCCGATCACGATGCGTTTCC-3′ and 5′-CTGTGCTTGACGGTTTGCTATCC-3′. Rn is the ratio between the fluorescence of the reporter dye and the fluorescence of the passive reporter dye ROX. Delta Rn (∆Rn) is calculated as Rn minus the baseline.

### Statistical analysis

The correlation coefficient *r* was calculated by Pearson’s analysis, applied to the measurement of the strength and direction of the linear relationship between each probe’s GC content and its cumulative sequencing coverage. Statistical analyses were performed using the JMP software, version 11 (SAS Institute, Cary, NC).

## Additional Information

**How to cite this article**: Miyazato, P. *et al*. Application of targeted enrichment to next-generation sequencing of retroviruses integrated into the host human genome. *Sci. Rep.* **6**, 28324; doi: 10.1038/srep28324 (2016).

## Supplementary Material

Supplementary Table S1

Supplementary Table S2

## Figures and Tables

**Table 1:** Number of reads obtained before and after the enrichment of naked DNA samples.

Sample	Enrichment (−)			Enrichment ( + )			Fold enrichment**
	Number of reads		% Ratio*	Number of reads		% Ratio*	
	Total	Virus-specific		Total	Virus-specific		
A	116,730,847	2,195,874	1.9	2,299,310	2,288,640	99.54	52.39
B	123,971,179	2,350,983	1.9	2,507,806	2,492,989	99.41	52.32

“%Ratio” (*) and “Fold enrichment” (**) were calculated as detailed in the Methods section.

**Table 2:** Number of reads obtained before and after the enrichment of ChIP DNA samples.

Sample	Enrichment (−)			Enrichment ( + )			Fold enrichment**
	Number of reads		% Ratio*	Number of reads		% Ratio*	
	Total	Virus-specific		Total	Virus-specific		
H3K4me3	57,478,945	1,206	0.0021	609,924	12,423	2.037	970
H3K9ac	57,995,401	330	0.0006	509,182	5,009	0.984	1640
H3K36me3	87,006,052	687	0.0008	3,684,872	64,507	1.751	2189
CTCF	59,855,472	172	0.0003	2,573,724	5,079	0.197	657
Naked DNA	17,839,510	29	0.00016	179,002	3,518	1.97	12313
Chromatin DNA	81,583,345	137	0.00017	747,261	17,051	2.281	13418

“% Ratio” (*) and “Fold enrichment” (**) were calculated as detailed in the Methods section.
